# Employment Status and Its Association With Psychological Distress and Alcohol Consumption

**DOI:** 10.7759/cureus.16054

**Published:** 2021-06-30

**Authors:** Namrata Walia, Rishtina Bhetuwal, Lianett Acosta, Swathi Muddasani, Chhaya Kamwal, Vivaswan D Mishra, David Leszkowitz

**Affiliations:** 1 Family Medicine, Baylor College of Medicine, Houston, USA; 2 Epidemiology, University of Texas School of Public Health, Houston, USA; 3 Medicine, Larkin Community Hospital, Miami, USA; 4 Medicine, Moti Lal Nehru Medical College, Allahabad, IND; 5 Family Medicine, Larkin Community Hospital, Miami, USA

**Keywords:** alcohol use, unemployment, anxiety, depression, psychological stresses, secondary data, stata

## Abstract

Purpose: The objective of this analysis was to find an association between employment status, psychological distress, and alcohol consumption.

Methods: Data from the 2020 Health Information National Trends Survey (HINTS) data was used. Patient health questionnaire-4 (PHQ-4) data and an average number of drinks per week were used to assess psychological distress and drinking status.

Results: Out of the 3865 people who answered the survey in the year 2020, 1890 (59.11%) were employed in one or multiple jobs during the time of the survey. The sample included 1561 men and 2204 women with an average age of 48.4 years. More than half (58.7%) of them were Non-Hispanic White followed by Hispanic and Non-Hispanic Black at 15.73% and 10.32%, respectively. Bivariate analysis showed a significant association between employment, psychological distress (p value=0.032), and alcohol drinking (p value=0.002); 60.59% of participants reporting severe distress (PHQ-4 score of 9-12) were unemployed. Alternatively, 73.1% of the employed respondents reported no stress (PHQ-4 score of 0-2). While more than half (75.97%) of those who were unemployed consumed only 1-2 drinks per week on an average, 7.27% consumed >13 drinks per week on an average. After adjusting for covariates, the regression analysis showed a highly significant association (p value< 0.001) between unemployment and psychological distress (OR=1.55; 95% CI 1.03, 2.31), and alcohol consumption (OR=0.67; 95% CI 0.48, 0.92).

Conclusion: Unemployment is associated with outcomes like psychological distress and alcohol consumption. However, being employed was found to be more strongly associated with alcohol drinking. We do not know if the coronavirus disease 2019 (COVID-19) pandemic was a risk factor for the given outcomes.

## Introduction

Severe acute respiratory syndrome coronavirus 2 (SARS-CoV-2) or more commonly known as coronavirus disease 2019 (COVID-19), was declared to be a public health emergency of international concern (PHEIC) by the World Health Organization (WHO) in early 2020 [1]. The pandemic has had a significant effect on the global economy leading to a sudden change in the employment status of several people. The increase in unemployment has been a major concern for the United States since the shutdown of the country due to the on-going pandemic. A total of 20.6 million people were reported to have lost their jobs in the first couple months of the pandemic in 2020, resulting in an unemployment rate of 14.7%, which gradually increased to 14.8% in April 2020 before it started to decline to 6.7% in October 2020 [2,3]. The rate of unemployment peaked in April 2020 during which nearly 49 million Americans were unemployed [4]. The highest rates of unemployment were reported in New Jersey (10.2%), followed by Hawaii (10.2%), Nevada (10.1%), New York (8.4%), and Louisiana (8.3%) [5].

The COVID-19 pandemic has increased the concerns about mental distress and substance use. Psychological distress, according to American Psychiatry Association (APA), is a set of painful mental and physical symptoms associated with normal fluctuations of mood. It can be assessed using specific clinical tools and can identify the beginning of common mental health issues such as anxiety and depression. If left untreated, it may lead to mental health conditions like major depressive disorder, anxiety disorder, schizophrenia, somatization disorders, sleep disorders, and several chronic health conditions [6,7]. McLachlan and Gale [7] showed that psychological distress significantly increases the risk of several other chronic health conditions like cardiovascular diseases, arthritis, chronic obstructive pulmonary disease.

Several risk factors for psychological distress have been reported in the literature. Some of the common ones include stressful life events and family history of depression, female gender, any other comorbid medical conditions, unemployment, immigration, academic achievements and or bullying experience at school, subjective memory complaint (SMC) [8-11]. Anxiety and depression are more common in the general population. An ultra-brief screening scale [12], patient health questionnaire-4 (PHQ-4), a four-point Likert-type scale was developed to measure the symptoms of anxiety and depression. It is the combination of 2-item measure of core symptoms (PHQ-2) for depression and 2-item measure for anxiety (GAD-2). It is a quick and easy clinical tool to screen patients for symptoms of anxiety or depression. Patients scoring high on this tool are assessed further for a definitive clinical diagnosis.

The pandemic has seen an increase in overall alcohol consumption by individuals [13]. According to the Dietary guidelines for Americans, provided by the U.S. Department of Health and Human Services and the U.S. Department of Agriculture [14], adult men should limit alcohol intake to a maximum of two drinks a day, whereas adult women should have no more than one alcoholic drink a day to lead a healthy, balanced life. Heavy alcohol use is defined as four or more drinks daily, or more than 14 drinks weekly for adult men. However, more than three alcoholic drinks daily, or more than seven drinks per week is considered heavy alcohol use for women. The National Institute on Alcohol Abuse and Alcoholism (NIAAA) defines binge drinking as an alcohol consumption typically five or more drinks for men, or four or more drinks for women during a time period of two hours. As per the Substance Abuse and Mental Health Services Administration (SAMHSA), binge drinking is also defined as five drinks for men, and four drinks per woman during a few hours, at least monthly [15]. Binge drinking is also defined as alcohol consumption that brings blood alcohol concentration to 0.08%.

A study performed in a large public University in Ohio focused on analyzing the alcohol consumption of college students after campus closure at the onset of the COVID-19 pandemic. The study also focused on the relationship between psychological distress, social support and changes in alcohol intake. It was discovered that campus closure during this pandemic led to an increase in alcohol consumption throughout, and those with anxiety and depression had a higher increase than those without any psychological distress [16]. Another study that focused on alcohol consumption in patients with a known history of alcohol use disorder was performed in the UK. The COVID-19 pandemic was a risk factor in itself for increased alcohol use, those with a history of alcohol use disorder were at a higher risk of relapse. In this study, there was an increase in alcohol consumption in 21% of the participants [13].

While many studies have reported the association of employment with alcohol consumption, several others have reported unemployment being associated with increase in alcohol intake. Psychological distress, on the other hand, has been reported to be only associated with unemployment. Moreover, alcohol intake and psychological distress have been shown to have a bi-directional association. The aim of this study is the examine the association of employment status (exposure) with alcohol consumption (outcome) and psychological distress (outcome) together.

## Materials and methods

Data collection

Data was obtained from the Health Information National Trends Survey (HINTS 5 Cycle 4) conducted in the year 2020 [17]. This national surveillance survey is conducted annually by the National Cancer Institute. The sample design for the survey consisted of two stages. An equal probability sample of addresses was selected from each sampling stratum in the first stage. The second stage consisted of one adult per sampled household. The sampling frame for Cycle 4 consisted of a single-mode mail survey using the Next Birthday Method for the respondent section. A total of 3865 respondents completed the survey questionnaire in this cycle. The completion rate (at least 80% survey completed) was 98.1% (n=3792).

No Institutional Review Board (IRB) approval was required to use a publicly available dataset. 

Measures

The survey asked the question on employment status: Which of the following best describe your current occupational status? Employed full time, employed part time, homemaker, student, retired, disabled, unemployed less than one year, unemployed one year or more, others. The answers were re-categorized into employed (full time and part time) and unemployed.

Psychological distress was assessed using a combined score from four separate questions. The question included in the survey was: Over the past two weeks, how often have you been bothered by: little interest, hopelessness, nervousness, and worrying. The answers included not at all, several days, more than half days, nearly every day. A new ordinal variable was created to interpret the total calculated PHQ-4 score for all four symptoms. The new variable phq4_score_interpret was categorized based on total PHQ-4 score interpretation as 0-2 as none; 3-5 as mild; 6-8 as moderate; and 9-12 as severe psychological distress. Alcohol consumption was calculated using the average number of weekly drinks. A new ordinal variable was created to convert the responses into categories of 1-2, 3-5, 6-8, 9-12, and more than equal to 13. 

Data analysis

The data was analyzed with STATA software, version 16.1 (Stata Corporation, College Station, TX, USA), using descriptive statistics and logistic regression analysis. To evaluate the association of unemployment with psychological distress and alcohol consumption, a weighted sample was used. A weighted sample better represents the general US adult population and adjusts for possible confounders in the sampling method. After controlling for potential confounders, logistic regression analysis was conducted. 

## Results

Table 1 shows the descriptive statistics for the sample population (with weighted proportions). The sample included 1561 men and 2204 women with a mean age of 48.4 years. More than half (58.7%) of the sample were Non-Hispanic White followed by Hispanic and Non-Hispanic Black at 15.73% and 10.32%, respectively. Around 15% of the respondents had an annual household income of less than $20,000 and 8% did not complete high school. In addition, more than half of the respondents had an annual household income of over $50,000 (60.72%) and had completed some college or beyond (69.14%). 1890 (59.11%) of the respondents were employed in one or multiple jobs during the time of the survey.

**Table 1:** Descriptive characteristics of the study sample with weighted percentages

Variables	n (weighted %)
Age (Mean, SD)	48.4 (0.30)
Gender	
Female	2204 (50.22%)
Male	1561 (47.57%)
Missing data	100 (2.21%)
Race and Ethnicity	
Non-Hispanic White	2133 (58.7%)
Non-Hispanic Black	596 (10.32%)
Non-Hispanic Asian	161 (4.6%)
Hispanic	481 (15.73%)
Others	119 (10.65%)
Education	
Less than High school	273 (8.03%)
High school graduate	705 (22.51%)
Some college	1081 (39.12%)
College graduate or more	1663 (30.02%)
Missing data	143 (0.32%)
Household income	
Less than $20,000	624 (15.15%)
$20,000 to $35,000	451 (11.46%)
$35,000 to $50,000	460 (12.67%)
$50,000 to $75,000	592 (18.26%)
$75,000 or more	1321 (42.46%)
Employment status	
Employed	1890 (59.11%)
Unemployed	1888 (40.89%)

Association of unemployment and psychological distress

Bivariate analysis in Table 2 showed significant association between employment status and psychological distress (p value=0.032). 77.5% (n=1268) of the unemployed respondents reported having no distress (PHQ-4 score of 0) while 14.5% reported having moderate to severe distress. 60.59% of participants reporting severe distress (PHQ-4 score of 9-12) were unemployed.

**Table 2:** Bivariate analysis of employment status and psychological distress PHQ-4: patient health questionnaire-4

	Psychological distress (PHQ-4 score)				P value
	None (0-2)	Mild (3-5)	Moderate (6-8)	Severe (9-12)	
Age					0.000*
Gender					0.159
Male	35.34%	8.15%	2.93%	2.82%	
Female	33.55%	9.41%	4.76%	3.04%	
Race and Ethnicity					0.0082*
Non-Hispanic White	44.22%	11.01%	4.99%	3.37%	
Non-Hispanic Black	7.99%	1.86%	0.36%	0.85%	
Non-Hispanic Asian	10.48%	3.47%	1.78%	0.96%	
Hispanic	4.39%	0.63%	0.22%	0.05%	
Others	1.48%	0.98%	0.52%	0.43%	
Education					0.2161
Less than High school	5.01%	1.18%	0.95%	0.67%	
High school graduate	14.46%	4.19%	1.82%	1.73%	
Some college	26.66%	7.97%	2.93%	1.84%	
College or more	22.34%	4.37%	2.34%	1.55%	
Household income					0.0008*
Less than $20,000	7.55%	4.21%	1.73%	1.44%	
$20,000 to $35,000	7.63%	1.99%	1.12%	0.65%	
$35,000 to $50,000	8.35%	1.78%	1.57%	0.60%	
$50,000 to $75,000	12.66%	3.54%	0.85%	1.28%	
$75,000 or more	31.77%	6.43%	2.82%	2.05%	
Occupation					0.0320*
Employed	42.52%	9.57%	4.69%	2.7%	
Unemployed	26.06%	17.35%	8.03%	6.04%	
*statistically significant					

After adjusting for covariates, the logistic regression analysis showed a highly significant association of employment status with psychological distress. Alcohol consumption showed an insignificant association with psychological distress; hence, it was not included in the final model. Table 3 shows that the unemployed had 1.03-2.31 times the odds of having psychological distress compared to respondents who were employed (p value=0.033). Age was shown to be significantly associated (OR=0.97; 95% CI 0.96, 0.98; p value<0.001) with distress symptoms. Participants who reported an annual household income of more than 75,000$ had lower odds (OR=0.39; 95% CI 0.24, 0.62) of having psychological distress compared to the ones with an annual household income of less than 20,000$ (p<0.001). Additionally, Non-Hispanic Blacks had lower odds (OR=0.69; 95% CI 0.48, 0.99) of psychological distress compared to Non-Hispanic Whites (p=0.046).

**Table 3:** Multivariable regression predicting psychological distress (PHQ-4 score) PHQ-4: patient health questionnaire-4

	Psychological distress	
Variables	AOR (95% CI)	P value
Occupation		
Employed	Reference	
Unemployed	1.55 (1.03, 2.31)	0.033*
Age	0.97 (0.96, 0.98)	0.000*
Household income		
Less than $20,000	Reference	
$20,000 to < $35,000	0.56 (0.35, 0.89)	0.015*
$35,000 to < $50,000	0.52 (0.29, 0.93)	0.029*
$50,000 to < $75,000	0.47 (0.29, 0.93)	0.008*
$75,000 or more	0.39 (0.24, 0.62)	0.000*
Race/Ethnicity		
Non-Hispanic White	Reference	
Non-Hispanic Black	0.69 (0.48, 0.99)	0.046*
Non-Hispanic Asian	0.99 (0.68, 1.43)	0.968
Hispanic	0.46 (0.23, 0.90)	0.024*
Others	1.94 (0.89, 4.19)	0.089
*statistically significant		

Association of unemployment and alcohol consumption

Bivariate analysis in Table 4 showed significant association between employment status and alcohol drinking (p value=0.002). While more than half (75.97%) of those who were unemployed consumed only 1-2 drinks per week on an average, 7.27% consumed >13 drinks per week on an average. Overall, out of 7.77% participants who reported heavy drinking (>13 drinks per week), more than half were employed. 

**Table 4:** Bivariate analysis of employment status and alcohol consumption

	Alcohol consumption (# of drinks per week)					P value
	1-2 drinks	3-5 drinks	6-8 drinks	9-12 drinks	>13 drinks	
Age						0.45
Gender						0.0001*
Male	31.12%	5.43%	3.31%	2.56%	6.31%	
Female	38.4%	5.25%	3.1%	2.07%	2.44%	
Race and Ethnicity						0.0042*
Non-Hispanic White	41.1%	7.89%	4.74%	3.93%	6.86%	
Non-Hispanic Black	8.4%	1.09%	0.48%	0.31%	0.37%	
Non-Hispanic Asian	12.87%	0.98%	0.84%	0.46%	1.2%	
Hispanic	4.02%	0.59%	0.31%	0.0%	0.16%	
Others	2.52%	0.31%	0.18%	0.0%	0.37%	
Education						0.0003*
Less than High school	6.04%	0.23%	0.27%	0.22%	0.67%	
High school graduate	14.9%	1.72%	1.23%	0.38%	2.79%	
Some college	29.14%	4.11%	2.36%	1.56%	3.01%	
College or more	19.22%	4.67%	2.61%	2.48%	2.38%	
Household income						0.0039*
Less than $20,000	11.07%	1.02%	0.72%	0.29%	0.08%	
$20,000 to $35,000	8.54%	0.68%	0.49%	0.31%	1.26%	
$35,000 to $50,000	8.66%	0.99%	0.62%	0.65%	1.09%	
$50,000 to $75,000	13.6%	2.0%	1.16%	0.54%	1.29%	
$75,000 or more	27.16%	5.91%	3.52%	2.91%	4.72%	
Occupation						0.0002*
Employed	39.1%	7.5%	4.36%	3.35%	6.03%	
Unemployed	30.41%	3.07%	2.06%	1.25%	2.87%	
*statistically significant						

After adjusting for covariates, the logistic regression analysis in Table 5 showed a highly significant association of unemployment and alcohol consumption (p=0.016). In addition, psychological distress was included in the final model. The unemployed respondents had 0.48-0.92 times the odds of consuming alcohol compared to the ones who were employed. Being a non-Hispanic Black (OR=0.53; 95% CI 0.32, 0.88) and women (OR=0.62; 95% CI 0.44, 0.89) had lower odds of alcohol consumption compared to non-Hispanic White and men. People with an annual household income of over 75,000$ were at 1.01-2.96 times the odds of alcohol consumption compared to the ones with a household income of less than 20,000$ (p=0.044).

**Table 5:** Multivariable regression predicting alcohol consumption (average weekly drinks)

Alcohol consumption (number of drinks per week)		
Variables	AOR (%CI)	P value
Occupation		
Employed	Reference	
Unemployed	0.66 (0.48, 0.90)	0.009^*^
PHQ-4 score		
None (0-2)	Reference	
Mild (3-5)	1.09 (0.74, 1.58)	0.664
Moderate (6-8)	0.80 (0.42, 1.53)	0.500
Severe (9-12)	1.85 (1.04, 3.31)	0.038^*^
Gender		
Male	Reference	
Female	0.61 (0.44, 0.83)	0.003^*^
Race/Ethnicity		
Non-Hispanic White	Reference	
Non-Hispanic Black	0.47 (0.31, 0.73)	0.001^*^
Non-Hispanic Asian	0.47 (0.31, 0.70)	0.000^*^
Hispanic	0.36 (0.17, 0.75)	0.007^*^
Others	0.47 (0.18, 1.28)	0.138
Household income		
Less than $20,000	Reference	
$20,000 to < $35,000	1.38 (0.73, 2.60)	0.307
$35,000 to < $50,000	1.37 (0.74, 2.40)	0.323
$50,000 to < $75,000	1.11 (0.65, 1.87)	0.703
$75,000 or more	1.70 (1.00, 2.85)	0.048^*^
*statistically significant		

## Discussion

Unemployment has been a major concern in the United States since 2020. The rate of unemployment was drastically influenced by the COVID-19 pandemic, and therefore the level of psychological distress. Using PHQ-4, the symptoms of anxiety and depression were measured. Results showed that levels of psychological distress were higher in those who were unemployed. Table 3 shows that unemployed individuals had higher odds of having psychological distress compared to those who were employed. Alcohol consumption, measured as the number of drinks per week, was shown to be higher in employed participants compared to the unemployed ones. This is contrary to other published studies in the literature that show association of unemployment with higher alcohol consumption. We cannot ascertain if the results of this study were influenced by the ongoing COVID-19 pandemic. However, several studies also showed alcohol consumption to be higher in those with higher education and higher annual income [18].

Several studies have published an association between employment status and psychological stress, or alcohol consumption. Paul and Moser [19] conducted a meta-analysis and reported an average of 34% of cases of psychological disorders among the unemployed compared to 16% in the employed individuals. In addition, the reported mental health outcomes due to unemployment were found to be more pronounced in countries with poor economic development [19]. Another study by Frasquilho et al. [20] conducted a questionnaire-based survey on 748 unemployed people to detect the association between economic and non-economic factors and psychological distress. The results showed the participant's mean distress was 46.5% higher than the national mean of Portugal. Several other studies have shown consistent results in various parts of the world [21-23]. According to a randomized household survey conducted in Montreal in 2009, employment status under 45-year-old is inversely related to the distress and depression, using coping strategies like social support and alcohol usage [24]. In a study conducted using a sample taken from Panel Study of Income Dynamics [25], they estimated the association between estimated income tax credit (EITC) and income and other outcomes of interest like smoking, alcohol intake, physiological distress, and overall health. They found that people with high income are associated with less physiological distress, more alcohol consumption, more cigarette smoking.

Anxiety, depression, and alcohol consumption are interlinked. Many individuals consume alcohol to reduce anxiety, and often, long-term alcohol abuse can lead to anxiety development [26]. Similarly, people with depression are more likely to abuse alcohol to suppress their symptoms and use drinking as a way to escape reality [27]. Therefore, it is critical to reach out to a primary care or mental health provider to reduce the worsening of these outcomes and their subsequent effect on daily life. SAMHSA provides various resources to address alcohol and mental health disorders including anxiety and depression. Campaigns like “National Prevention week” which takes place in May, encourages community involvement, shares resources, and aims to increase public awareness on substance abuse including alcohol use [28]. There are several other organizations such as Centerstone, that distribute educational resources including alcohol-related statistics from published research, fact sheets, and videos that cover the effects of heavy drinking and how to recognize drinking problems [29].

Study limitations

There are several limitations to this study. First, the HINTS dataset did not ask questions on alcohol consumption in the past 12 months. Because of the lack of baseline data on alcohol consumption, a change over time could not be assessed. Moreover, since this dataset is a cross-sectional survey design, it is not possible to infer causal relationship between the variables. Second, a two-way relationship between alcohol use or psychological distress and unemployment could not be established due to the study design. Such associations are best established using prospective study designs. Lastly, the survey data used for this study was conducted in 2020. There was no efficient measure to adjust the confounding effect of the ongoing COVID-19 pandemic on the outcome variables. 

## Conclusions

Unemployment is associated with outcomes like psychological distress and alcohol consumption. However, being employed was found to be more strongly associated with alcohol drinking. We do not know if the COVID-19 pandemic was a risk factor for the given outcomes. Federal aid through the Coronavirus Aid, Relief, and Economic Security (CARES) Act 2020 provided compensation to workers who became unemployed due to the COVID-19 pandemic. In addition, mutual-support groups like the alcoholics anonymous (AA) program or pharmacological and psychotherapy treatment for those experiencing anxiety or depressive symptoms are effective options to seek. Psychotherapy focuses on thoughts, feelings, and issues that are happening in an individual's life. It teaches them skills to help cope with their life, change behaviors that are causing problems, and find solutions.

## References

[1] (2021). WHO coronavirus (COVID-19) dashboard. http://covid19.who.int/.

[2] Amadeo Amadeo, K. (2021, May 10 (2021). What is the current US unemployment rate?. https://www.thebalance.com/current-u-s-unemployment-rate-statistics-and-news-3305733.

[3] (2021). US job losses due to COVID-19 highest since Great Depression. https://www.cidrap.umn.edu/news-perspective/2020/05/us-job-losses-due-covid-19-highest-great-depression.

[4] Fronstin P, Woodbury SA (2020). How many Americans have lost jobs with employer health coverage during the pandemic?. Commonwealth Fund.

[5] (2021). Congressional Research Service: unemployment rates during the COVID-19 pandemic (R46554). https://crsreports.congress.gov/product/details?prodcode=R46554.

[6] (2021). American Psychological Association: APA dictionary of psychology. https://dictionary.apa.org/psychological-distress.

[7] McLachlan KJJ, Gale CR (2018). The effects of psychological distress and its interaction with socioeconomic position on risk of developing four chronic diseases. J Psychosom Res.

[8] Zilber N, Lerner Y (1996). Psychological distress among recent immigrants from the former Soviet Union to Israel, I. Correlates of level of distress. Psychol Med.

[9] Myklestad I, Røysamb E, Tambs K (2012). Risk and protective factors for psychological distress among adolescents: a family study in the Nord-Trøndelag Health Study. Soc Psychiatry Psychiatr Epidemiol.

[10] Paradise MB, Glozier NS, Naismith SL, Davenport TA, Hickie IB (2011). Subjective memory complaints, vascular risk factors and psychological distress in the middle-aged: a cross-sectional study. BMC Psychiatry.

[11] Achdut N, Refaeli T (2020). Unemployment and psychological distress among young people during the COVID-19 pandemic: psychological resources and risk factors. Int J Environ Res Public Health.

[12] Kroenke K, Spitzer RL, Williams JB, Lowe B (2009). An ultra-brief screening scale for anxiety and depression: the PHQ-4. Psychosomatics.

[13] Kim JU, Majid A, Judge R (2020). Effect of COVID-19 lockdown on alcohol consumption in patients with pre-existing alcohol use disorder. Lancet Gastroenterol Hepatol.

[14] (2021). Dietary guidelines for Americans. https://www.dietaryguidelines.gov/.

[15] (2021). NIH: drinking levels defined. http://www.niaaa.nih.gov/alcohol-health/overview-alcohol-consumption/moderate-binge-drinking.

[16] Lechner WV, Laurene KR, Patel S, Anderson M, Grega C, Kenne DR (2020). Changes in alcohol use as a function of psychological distress and social support following COVID-19 related university closings. Addict Behav.

[17] (2021). Health Information National Trends Survey (HINTS) dataset. https://hints.cancer.gov/.

[18] Collins SE (2016). Associations between socioeconomic factors and alcohol outcomes. Alcohol Res.

[19] Paul KI, Moser K (2009). Unemployment impairs mental health: meta-analyses. J Vocat Behav.

[20] Frasquilho D, de Matos MG, Marques A, Gaspar T, Caldas-de-Almeida JM (2016). Distress and unemployment: the related economic and noneconomic factors in a sample of unemployed adults. Int J Public Health.

[21] Arias-de la Torre J, Fernández-Villa T, Molina AJ (2019). Psychological distress, family support and employment status in first-year university students in Spain. Int J Environ Res Public Health.

[22] Sidorchuk A, Engström K, Johnson CM, Kayser Leeoza N, Möller J (2017). Employment status and psychological distress in a population-based cross-sectional study in Sweden: the impact of migration. BMJ Open.

[23] Stankunas M, Kalediene R, Starkuviene S, Kapustinskiene V (2006). Duration of unemployment and depression: a cross-sectional survey in Lithuania. BMC Public Health.

[24] Perreault M, Touré EH, Perreault N, Caron J (2017). Employment status and mental health: mediating roles of social support and coping strategies. Psychiatr Q.

[25] Shields-Zeeman L, Collin DF, Batra A, Hamad R (2021). How does income affect mental health and health behaviours? A quasi-experimental study of the earned income tax credit. J Epidemiol Community Health.

[26] Edited by Meredith Watkins, M. (2021, April 29 (2021). How anxiety and alcohol are linked. https://americanaddictioncenters.org/alcoholism-treatment/anxiety.

[27] (2021). The link between alcohol abuse and depression. https://www.alcoholrehabguide.org/resources/dual-diagnosis/alcohol-and-depression.

[28] (2021). Prevention of substance use and mental disorders. https://www.samhsa.gov/find-help/prevention#substance-use-disorder-prevention.

[29] (2021). Mental health conditions: depression and anxiety. https://www.cdc.gov/tobacco/campaign/tips/diseases/depression-anxiety.html.

